# Temporal and Geographical Disparities in Amyloid and Heart Failure-Related Mortality: An Epidemiological Study 1999-2020

**DOI:** 10.2174/011573403X374069250630105650

**Published:** 2025-07-11

**Authors:** Hoang Nhat Pham, Ramzi Ibrahim, Thi Nguyen, Enkhtsogt Sainbayar, Mahek Shahid, João Paulo Ferreira, Amitoj Singh, Kwan Lee, W. H. Wilson Tang, Preethi William

**Affiliations:** 1 Department of Medicine, University of Arizona, Tucson, AZ 85721, United States;; 2 Department of Cardiovascular Medicine, Mayo Clinic, Phoenix, AZ 85054, United States;; 3 Department of Cardiovascular Medicine, Heart Vascular and Thoracic Institute, Cleveland Clinic, Cleveland, OH 44106, United States

**Keywords:** Amyloid, heart failure, infiltrative, age-adjusted mortality rates, monte-carlo permutation, log-linear regression models

## Abstract

**Introduction:**

Amyloidosis complicated by heart failure (HF) poses significant mortality. We sought to identify trends in comorbid amyloidosis and HF mortality in the recent 22-year period.

**Methods:**

Mortality due to amyloid and HF as contributors of death were queried from death certificates using the CDC database from 1999 to 2020. Mortality rates and their 95% confidence intervals were adjusted for age (AAMR) through the Direct method and compared by demographic subpopulations. The Monte-Carlo permutation test was used to estimate the annual percentage change (APC). Log-linear regression models were utilized to assess temporal variation in mortality.

**Results:**

Age-adjusted mortality rates (AAMR) increased from 0.09 (0.08-0.10) in 1999 to 0.27 (0.25-0.29) in 2020. Mortality increased from 1999 to 2013 (APC +1.4, *p=*0.048) with an accelerating inflection point in 2013 to 2020 (APC +13.3, *p<*0.001). AAMR was higher among male populations (AAMR 0.20 [0.20-0.21]) compared to female populations (AAMR 0.07 [0.07-0.07]). A significant inflection point in uprising mortality rates was observed for both male and female populations in 2013 (*p<*0.001). Mortality was highest among Black populations (AAMR 0.33), followed by White (AAMR 0.10), Asian/Pacific Islander (AAMR 0.06), and American Indian/Alaska Native populations (AAMR 0.04). Among Black populations, mortality remained consistent from 1999 to 2012 (APC +1.1, *p=*0.184), followed by an increase from 2012 to 2020 (APC +14.0, *p<*0.001). Among White populations, mortality remained stagnant from 1999 to 2013 (APC +0.7, *p=*0.302), followed by an increase starting in 2013 to 2020 (APC +13.5, *p<*0.001).

**Discussion:**

Our findings of a marked rise in HF-related mortality in patients with amyloidosis since 2013 highlighted the profound impact of enhanced diagnostic awareness, novel imaging techniques, and emerging therapeutics. Our analysis also showed mortality disparities between sexes, and geographic locations, races, and ethnicity that warrant targeted public health interventions.

**Conclusions:**

Amyloid and HF mortality increased in the recent 22-year period, primarily starting in 2013, emphasizing the urgent need for targeted intervention to address these disparities.

## 1 INTRODUCTION

Amyloidosis is a rare group of heterogeneous disorders caused by misfolded proteins that aggregate into insoluble fibrils and ultimately cause organ damage. To date, over 30 different subtypes have been identified, with 14 of them associated with systemic amyloidosis [1]. These proteins aggregate and form amyloid fibrils within various organs, including the heart, liver, kidney, and nervous system. Cardiac involvement plays a crucial role in the prognosis of amyloidosis, with over half of the patients succumbing to cardiac complications [2]. The deposition of amyloid fibrils in the heart results in the replacement of cardiac myocytes, leading to conduction malfunctions, ventricular thickening, ventricular diastolic dysfunction, and systolic dysfunction at its advanced stages [3].

The two most common types of cardiac-infiltrating amyloidosis are acquired immunoglobulin light chain (AL) amyloidosis and transthyretin (ATTR) amyloidosis. ATTR amyloidosis can be inherited through a mutation in the transthyretin protein (mATTR) or acquired (wild-type ATTR, wtATTR) in older individuals. AL light chain amyloidosis is the most frequently diagnosed form of cardiac amyloidosis, followed by acquired ATTR amyloidosis [4]. Despite often less severe wall thickening in AL amyloidosis compared to ATTR, the clinical course of AL amyloidosis is typically more aggressive [5]. The prognosis for cardiac ATTR amyloidosis is generally more favorable than that for cardiac AL amyloidosis, with a median survival of 3-5 years from diagnosis, compared to a median survival of 1.08 years for AL amyloidosis, which further reduces to 0.75 years with the onset of heart failure [5, 6]. Nonetheless, early diagnosis of cardiac amyloidosis remains a significant challenge due to its nonspecific clinical presentation, which often leads to delayed recognition. However, the emergence of diagnostic tools such as cardiac MRI and technetium 99m-3,3-diphosphono-1,2-propanodicarboxylic acid (99mTc-DPD) scintigraphy has facilitated earlier and more accurate diagnosis in recent years [7].

Heart failure is the leading cause of death in patients with cardiac amyloidosis, accounting for 64% of cardiovascular deaths [2, 8]. Despite the poor prognosis associated with cardiac amyloidosis and heart failure, it remains poorly recognized, especially among underrepresented populations [9-11]. To the best of our knowledge, limited studies on a United States (US) national representative sample has evaluated the trends in comorbid HF and amyloidosis related mortality. Therefore, we aimed to investigate the mortality rates of HF and amyloidosis across demographic subpopulations and explore temporal changes from 1999 to 2020.

## 2 MATERIALS AND METHODS

We utilized the Multiple Cause of Death files from the Centers for Disease Control and Prevention (CDC) Wide-ranging Online Data for Epidemiologic Research (WONDER) database from 1999 to 2020 [12, 13]. Using International Classification of Diseases, Tenth Revision (ICD-10) code queries, all deaths related to heart failure (ICD-10: I11.0, I13.0, I13.2, I150.0, I150.1, I150.9) and amyloidosis (E85.0, E85.1, E85.2, E85.3, E85.4, E85.8, E85.9) as contributing causes of death were ascertained. We also obtained relevant demographic information, including sex, race and ethnicity, and area of residence. Race and ethnicity were included in death certificates by report of a next-of-kin; however, in the absence of such a report, this was based solely on observation. Race was categorized as non-Hispanic Black, White, American Indian/Alaska Native, and Asian/Pacific Islander populations. Ethnicity was classified as Hispanic and non-Hispanic populations. Area of residence was categorized based on the US census area of residence (*i.e.*, Northeast, Midwest, South, and West) and the 2013 National Center for Health Statistics urbanization scheme (*i.e.*, metropolitan and non-metropolitan).

Mortality data were compiled from 57 vital statistics jurisdictions through the Vital Statistics Cooperative Program, based on death certificates for US residents. Total death counts were obtained for cumulative and demographic subpopulations. Crude mortality rates (CMR) were calculated by dividing the number of deaths by the corresponding population size. We adjusted mortality for age using the Direct method with the US population in 2000 as the standard. We tested for temporal variation in yearly age-adjusted mortality rates (AAMR per 100,000 population) using log-linear regression models (Joinpoint Regression; National Cancer Institute) [14]. Temporal changes in mortality for all populations except by ethnicity and two of the racial subgroups (*i.e.*, Asian/Pacific Islander and American Indian/Alaska Native) due to suppression of yearly mortality rates in the setting of low death counts and confidentiality purposes. We utilized the Monte-Carlo permutation test to estimate the annual percentage change (APC), and based on these averages, we calculated the average annual percentage change. To estimate increasing or decreasing trends in the AAMR, two-tailed t-test statistics were conducted. A two-tailed *p*-value of <0.05 was set for statistical significance.

De-identified data from the CDC WONDER database is publicly available. As the data was collected by the CDC for surveillance purposes, ethical approval and informed consent were not applicable to this secondary analysis and are exempt from Institutional Review Board approval.

## 3 RESULTS

**Fig. (1):**
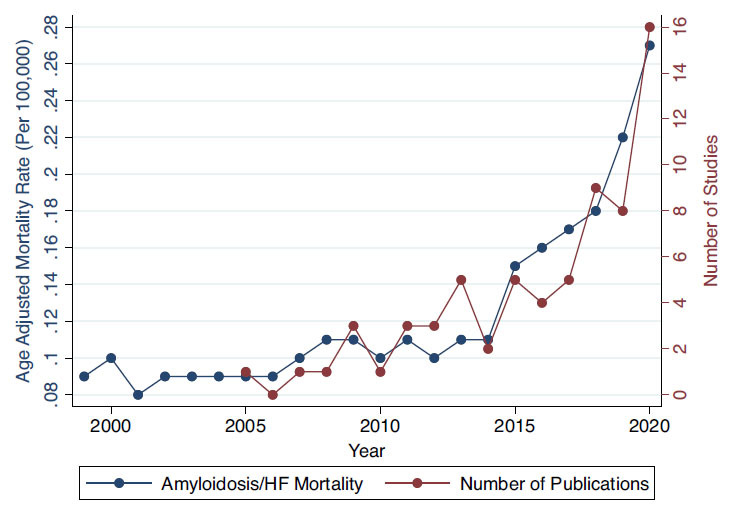
Annual HF/amyloidosis-associated mortality by number of related publications. Yearly HF and amyloidosis AAMR in the US from 1999 to 2020, plotted along the number published clinical cohorts or registries (in English language) related to cardiac amyloidosis (2005-2020). **Abbreviations:** AAMR = age-adjusted mortality rate, HF = heart failure, US = United States.

Between 1999 and 2020, a total of 9,526 deaths were attributed to both HF and amyloidosis. The AAMR increased from 0.09 (95% CI, 0.08-0.10) in 1999 to 0.27 (95% CI, 0.25-0.29), with a cumulative AAMR of 0.14 (95% CI, 0.14-0.14) (Fig. **1**). Mortality increased from 1999 to 2013 (APC +1.4%, *p=*0.048) with an accelerating inflection point in 2013 to 2020 (APC +13.3%, *p<*0.001).

**Fig. (2):**
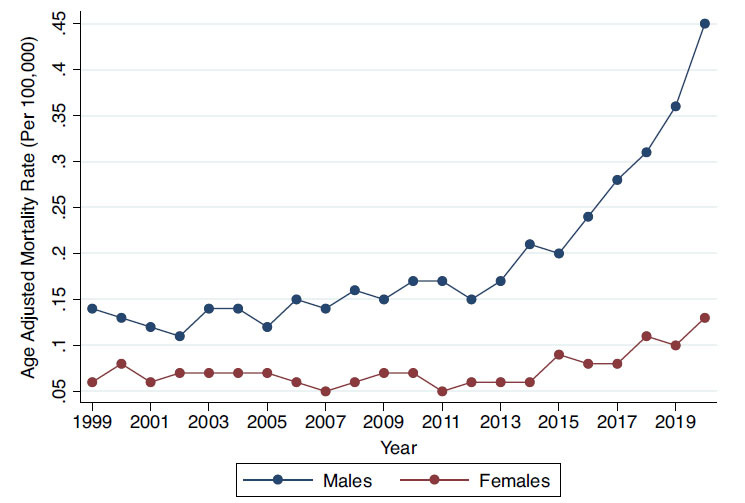
HF/Amyloidosis-associated mortality by sex. Yearly HF and amyloidosis AAMR in the US from 1999 to 2020, stratified by sex. **Abbreviations:** AAMR = age-adjusted mortality rate, HF = heart failure, US = United States.

The AAMR was higher among male populations (AAMR 0.20 [95% CI, 0.20-0.^21^]) compared to female populations (AAMR 0.07 [95% CI, 0.07-0.0^7^]). Mortality increased steadily among males from 1999 to 2013 (APC +0.8%, *p=*0.003) with an accelerating inflection point in 2013 to 2020 (APC +13.8%, *p<*0.001). Mortality among female populations remained stagnant from 1999 to 2013 (APC -1.0%, *p=*0.300), followed by an increase beginning in 2013 until 2020 (APC +10.8%, *p<*0.001) (Fig. **2**).

**Fig. (3):**
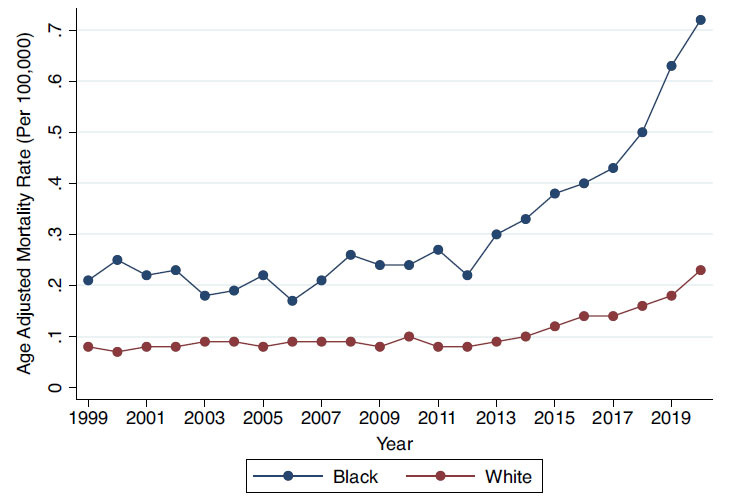
HF/Amyloidosis-associated mortality by race. Yearly HF and amyloidosis AAMR in the US from 1999 to 2020, stratified by race. **Abbreviations:** AAMR = age-adjusted mortality rate, HF = heart failure, US = United States.

Mortality was higher among non-Hispanic populations (AAMR 0.14 [95% CI, 0.14-0.^15^]) compared to Hispanic populations (AAMR 0.07 [95% CI, 0.06-0.0^8^]). Among the non-Hispanic populations, mortality was highest among Black populations (AAMR 0.33 [95% CI, 0.31-0.^35^]), followed by White (AAMR 0.10 [95% CI, 0.10-0.^10^]), Asian/Pacific Islander (AAMR 0.06 [95% CI, 0.05-0.0^7^]), and American Indian/Alaska Native populations (AAMR 0.04 [95% CI, 0.03-0.0^7^]). Mortality among Black populations remained consistent from 1999 to 2012 (APC +1.1%, *p=*0.184), followed by an increase in mortality from 2012 to 2020 (APC +14.0%, *p<*0.001). Among White populations, mortality remained stagnant from 1999 to 2013 (APC +0.7%, *p=*0.302), followed by an increase in mortality starting in 2013 to 2020 (APC +13.5%, *p<*0.001) (Fig. **3**).

**Fig. (4):**
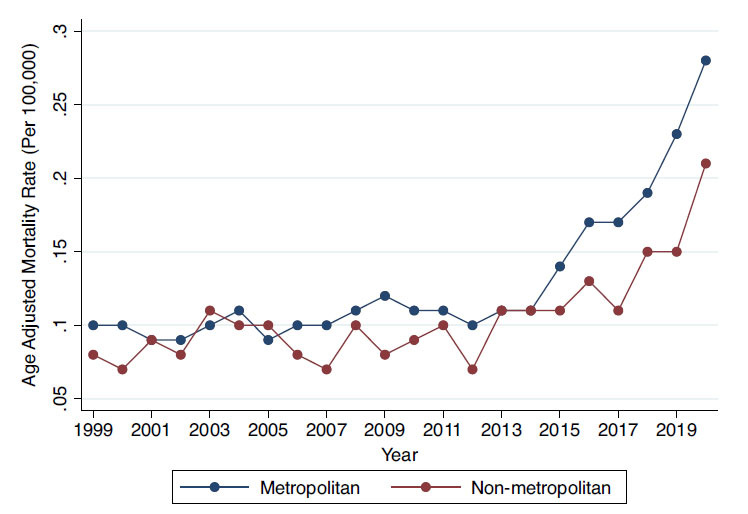
HF/Amyloidosis-associated mortality by urbanization. Yearly HF and amyloidosis AAMR in the US from 1999 to 2020, stratified by urbanization. **Abbreviations:** AAMR = age-adjusted mortality rate, HF = heart failure, US = United States.

Mortality was highest among metropolitan regions (AAMR 0.14 [95% CI, 0.14-0.^15^]) compared to non-metropolitan regions (AAMR 0.11 [95% CI, 0.10-0.^11^]). From 1999 to 2014, mortality within metropolitan regions was rising steadily (APC +1.3%, *p=*0.025), followed by a significant inflection point in 2014 (APC +15.5%, *p<*0.001). From 1999 to 2017, mortality among non-metropolitan regions increased steadily (APC +2.0%, *p=*0.008), followed by an inflection point in 2017 until 2020 (APC +22.0%, *p=*0.024) (Fig. **4**).

**Fig. (5):**
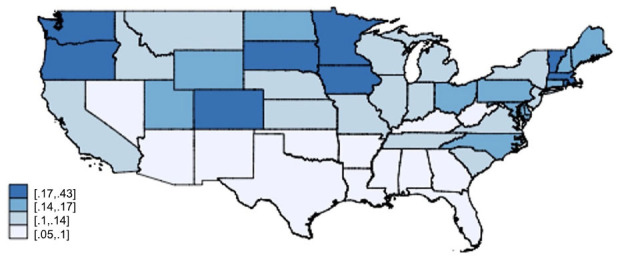
US choropleth map of HF/Amyloidosis-associated mortality. US map depicting all HF and amyloidosis mortality, stratified by state. **Abbreviations:** HF = heart failure, US = United States.

**Fig. (6):**
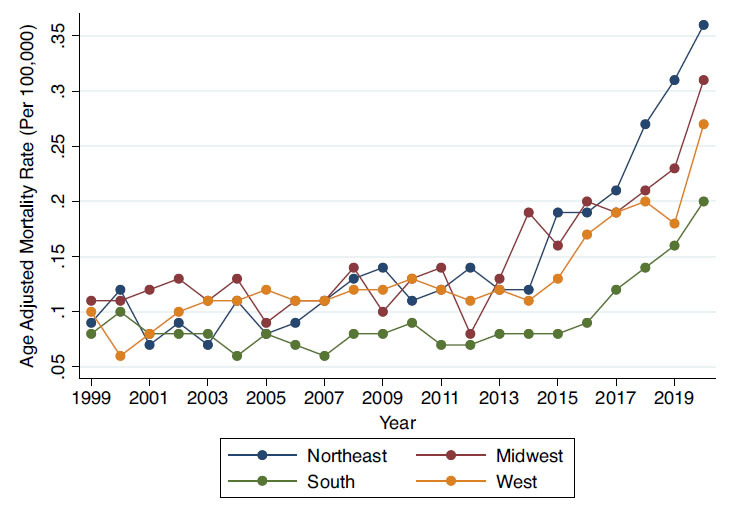
HF/amyloidosis-associated mortality by US census regions. Yearly HF and amyloidosis AAMR in the US from 1999 to 2020, stratified by US Census Regions. **Abbreviations:** AAMR = age-adjusted mortality rate, HF = heart failure, US = United States.

Mortality was similar among the Northeastern (AAMR 0.15 [95% CI, 0.14-0.^16^]), Midwestern (AAMR 0.15 [95% CI, 0.15-0.^16^]), and Western (AAMR 0.14 [95% CI, 0.14-0.^15^]) US regions, but lowest in the Southern regions (AAMR 0.08 [95% CI, 0.08-0.0^9^]). Mortality increased steadily in Northeastern US regions from 1999 to 2014 (APC +2.6%, *p=*0.008), followed by a significant inflection point in 2014 until 2020 (APC +18.4%, *p<*0.001). Mortality remained stagnant in Midwestern US regions from 1999 to 2012 (APC +0.5%, *p=*0.610), followed by an accelerating inflection point in 2012 until 2020 (APC +11.1%, *p<*0.001). Similarly, mortality rates in Southern US regions remained stagnant from 1999 to 2015 (APC -0.4%, *p=*0.484), followed by an accelerating inflection point in 2015 (APC +21.1%, *p<*0.001). Among Western US regions, mortality increased steadily from 1999 to 2014 (APC +1.9%, *p<*0.040), followed by an inflection point in 2014 until 2020 (APC +10.7%, *p<*0.001) (Figs. **5** and **6**).

## 4 DISCUSSION

In this study, we aimed to investigate the temporal and regional mortality trends of decedents related to amyloidosis and HF among populations of various sexes, races/ethnicities, and areas of residence in the US. Our findings revealed that annual amyloid and HF-related AAMR increased in the recent 22-year period, with a significant rise beginning in 2013 and 2014. This pattern remained consistent cumulatively and across the subpopulations. A higher mortality rate was observed in males compared to females, in non-Hispanic populations compared to Hispanic populations, and in metropolitan areas. These findings provide insightful epidemiological data on amyloidosis and HF-related mortality in the US in the recent 22-year period.

Our study evaluated amyloid and HF related mortality trends during a time period that coincided with the widespread adoption of new imaging modalities for cardiac amyloidosis in early 2010s, the publication of ATTR-ACT (Tafamidis Treatment for Patients with Transthyretin Amyloid Cardiomyopathy) trial in 2018, and the Food and Drug Administration (FDA) approval of Tafamidis in 2019 [15-17]. Consistently, we observed increasing mortality trends associated with amyloidosis and HF, primarily starting in 2013. The recent advances in diagnostic and therapeutic modalities for cardiac amyloidosis have led to increased recognition of this condition as well as better disease detection, starting primarily in 2005, followed by the introduction of the Technetium Tc 99m Pyrophosphate and 99m Tc-DPD bone scintigraphy in early 2010. These findings coincide with the discovery of the relative “apical sparing” pattern of longitudinal strain on 2-D speckle tracking in 2012, which then led to broad clinical adoption of strain imaging and PYP scans, further contributing to improved disease detection [16, 18-20]. Concurrently, the ATTR-ACT trial, enrolling patients from December 2013 to August 2015, resulted in the FDA approval of tafamidis in 2019, due to its demonstrated efficacy in the reduction of mortality, hospitalizations, and functional decline [15]. These developments correspond with the findings of Zampieri *et al*., who reported a marked increase in amyloidosis diagnoses between 2000 and 2019, particularly with an exponential growth in wild-type ATTR (wtATTR) [21]. This heightened disease awareness is also reflected in the rise of publications on clinical outcomes of cardiac amyloidosis [22, 23] (Fig. **6**). Another contributing factor to the increasing mortality trends seen in our analyses may be related to the aging of the population, which has been shown to be an independent predictor of increased cardiac amyloidosis mortality [24]. This contrasts with the Heart Failure Epidemiology and Outcomes Statistics, which report a greater relative annual increase in heart failure-related mortality rates among younger adults compared to their older counterparts in recent years [25].

Our results revealed a higher overall amyloidosis and HF-related AAMR among male populations compared to female populations, contradictory to previous reports [23]. This is consistent with the high prevalence of wtATTR, associated with poor prognosis, in older White males [26]. Furthermore, the prevalence of wtATTR diagnoses had increased exponentially over the last 20 years; while it was nearly undiagnosed before 2010, it accounted for 73% of all amyloidosis diagnoses by 2019 [21]. Within our included populations, heart failure ICD-10 codes did not differentiate between reduced or preserved ejection fractions. The undifferentiation in ejection fraction may have contributed to the sex indifferences in mortality rates, as males have historically been found to have progressive and more severe cardiac amyloidosis outcomes, especially in the setting of a reduced ejection fraction, whereas previous studies frequently included only preserved ejection fraction [5, 23, 27, 28]. Despite historical classification of amyloid heart disease as a form of HFpEF diagnosis, reduced LVEF has been reported in up to almost half of individuals with cardiac amyloidosis [29]. Furthermore, our findings related to racial and ethnic mortality disparities in patients with amyloidosis and HF revealed that non-Hispanic populations had the highest mortality rates, particularly affecting Black populations. This is consistent with previous findings related to cardiac amyloidosis outcomes [30]. For example, approximately 4% of black individuals carry the amyloidogenic transthyretin gene mutation, in which isoleucine is substituted for valine at position 142 (p.Val142Ile), historically reported as Val122Ile, leading to autosomal dominant cardiomyopathy with higher risk of HF and death [31].

Wide geographic variation in amyloidosis and HF mortality was seen in our results, likely a result of under-diagnosis in certain regions. These findings reflect the disparities in resource availability and access to healthcare [30, 32, 33]. For example, Amyloidosis Centers of Excellence are more concentrated in the Northeast, Midwest, and the West, while only one center exists in the Southern US [34]. Furthermore, our analysis also revealed higher mortality and an earlier uptrend in mortality rates within metropolitan regions, likely a component of diagnostic and therapeutic availability in urban regions [35].

This study has limitations that should be considered. Our analysis was undifferentiated between heart failure with reduced or preserved ejection fraction and between amyloidosis subtypes (AL, ATTR). Furthermore, use of ICD-10 codes to identify decedents of interest may lead to misclassification errors. However, amyloidosis has well-defined clinicopathological characteristics, decreasing the likelihood of falsely classifying a diagnosis as amyloid. Additionally, the concurrent ICD-10 codes of both amyloidosis and heart failure may have captured decedents that had concomitant disease processes without amyloidosis being the sole culprit for the heart failure. Despite these limitations, our analysis utilized a widely capturing database that is nationally representative and includes over 99% of decedents in the US [36]. Previous studies were limited to single-center populations or registries that were not as comprehensive [23, 28, 30, 37-39].

## CONCLUSION

In this nationally representative analysis spanning over two decades, we observed a significant and accelerating increase in amyloidosis and HF-related mortality in the United States, particularly after 2013. This upward trend was consistent across most demographic and geographic subpopulations, with notably higher mortality rates among males, the Black population, and those residing in metropolitan areas. While these findings may reflect improved detection and recognition of cardiac amyloidosis, they also highlight persistent disparities in disease burden, healthcare access, and outcomes. Although the limitations of our analysis warrant cautious interpretation, our study offers valuable insights into epidemiological understanding of the impact of amyloidosis on HF on a population level and emphasizes the urgent need for targeted intervention to address these disparities.

## AUTHORS’ CONTRIBUTIONS

The authors confirm their contribution to the paper as follows: study conception and design: HNP, RI, TN, ES, MS, JPF, AS, KL, WT and PW; data collection: HNP and RI; analysis and interpretation: HNP, RI, TN, ES, MS, JPF, AS, KL, WT and PW; draft manuscript: HNP, RI, TN, ES and MS. All authors reviewed the results and approved the final version of the manuscript.

## Data Availability

All the data and supporting information are provided within the article.

## LIST OF ABBREVIATIONS

AAMR: Age-Adjusted Mortality Rate
AL: Acquired Immunoglobulin Light Chain
ATTR: Transthyretin Amyloid
CDC: Centers for Disease Control and Prevention
HF: Heart Failure
ICD10: International Classification of Diseases, 10th Revision
US: United States
WONDER: Wide-ranging Online Data for Epidemiologic Research
